# Myeloid Cell Function and Cytokine Profiles in Paediatric Haemophilia A: Insights From FVIII and Emicizumab Prophylaxis

**DOI:** 10.1111/jcmm.71216

**Published:** 2026-05-27

**Authors:** Alessia Cottonaro, Saicharan Akula, Berardino Pollio, Irene Ricca, Tiziano Martini, Roberto Albiani, Jacopo Agnelli Giacchello, Patrizia Sciancalepore, Roberto Santi, Antonia Follenzi, Simone Merlin

**Affiliations:** ^1^ Health Sciences Università del Piemonte Orientale Novara Italy; ^2^ Diagnostic Department A.O. Città Della Salute e Della Scienza, SIMT Immunohematology and Transfusion Medicine Service Torino Italy; ^3^ Azienda Ospedaliera SS. Antonio e Biagio e Cesare Arrigo Alessandria Italy

**Keywords:** HA prophylaxis, myeloid cells, paediatric patients

## Abstract

Haemophilia A (HA) is an X‐linked bleeding disorder caused by factor VIII (FVIII) deficiency, treated with FVIII infusions or, more recently, Emicizumab subcutaneously. Although Emicizumab is safe and effective, FVIII is still required for severe bleeding, trauma, or surgery, and few studies have compared these prophylactic options in paediatric patients. This study explores the immunological and haematological profiles of paediatric HA patients receiving FVIII or Emicizumab, using haemophilia B patients and healthy controls. Clinical parameters and immune cell populations showed no major differences aside from age‐related variations. However, HA patients displayed higher HLA‐DR expression on CD14^+^ cells than healthy controls, and Emicizumab‐treated patients showed increased HLA‐DR expression on CD11c^+^ cells compared with FVIII‐treated patients. Plasma cytokines including IL‐12p40, TNF‐α, CCL‐22, IL‐18, and CCL‐4 were elevated in HA, suggesting a dysregulated myeloid compartment in HA. Patient‐derived macrophages exhibited a stronger pro‐inflammatory (M1‐like) polarization after in vitro FVIII stimulation, with increased TNF‐α and reduced TGF‐β gene expression. Stratification by prophylaxis showed that macrophages from FVIII‐treated patients maintained the M1 phenotype, whereas those from Emicizumab‐treated patients showed no clear shift and tended toward an immune‐regulatory profile. These findings highlight distinct myeloid and cytokine signatures associated with different prophylaxis, emphasizing the need for optimized therapeutic strategies.

## 1 Introduction

Haemophilia is an inherited X‐linked disorder characterized by reduced or absent activity of a coagulation factor (F) due to mutations or deletions in F8 or F9 genes, leading to haemophilia A (HA) and haemophilia B (HB) respectively [1, 2]. HA has a global prevalence of approximately 1 in 5000 male live births and its severity is classified based on residual plasma FVIII activity in: severe, when factor level is < 1%; moderate, when it is 1%–5%; mild, when the range is from 5% to 40% [3]. The main clinical manifestation is bleeding, either spontaneous or following a trauma [4], with the former typically affecting soft tissues, muscles and joints, leading to hematomas, hemarthrosis and synovial tissue inflammation that can eventually progress to hemophilic arthropathy [4] Standard HA treatment involves prophylactic FVIII replacement, though its efficacy is limited by a short half‐life – partially addressed by EHL molecules [5, 6, 7]– and the frequent development of neutralizing antibodies (inhibitors), which occur in 30% of severe HA typically within the first 50 exposure days [8, 9], affecting the efficacy of the replacement therapy. Currently, immune tolerance induction (ITI) remains the primary but limited solution for inhibitor eradication, with a 30% success rate [10]. Non‐replacement therapies first gained ground as ITI substitutes and include drugs that are able to restore haemostasis promoting thrombin generation with mechanisms different from exogenous FVIII infusions. Newer molecules such as the RNA interference therapeutic Fitusiran and the tissue factor pathway inhibitors have shown significant “rebalancing” potential and are promising therapeutic options [11]; Emicizumab, a humanized bispecific antibody able to bind activated FIX and FX, is currently a consolidated prophylactic therapy for HA patients of all ages, with or without inhibitor [12, 13]. Clinical trials, such as the HAVEN studies, have established its efficacy, safety profile, and its ability to significantly reduce the annual bleeding rate (ABR) in HA patients with or without inhibitors. Notably, the HAVEN 7 study was the first clinical trial to demonstrate the efficacy and tolerability of Emicizumab in infants (< 12 months old), opening new possibilities for very young patients [14]. Despite these advancements, there is limited research directly comparing the immune and clinical effects of recombinant FVIII (rFVIII) and Emicizumab in paediatric HA patients. It has been reported that inhibitor‐positive patients demonstrated an up regulation of innate immune modulator genes, suggesting that innate immunity plays a critical role in the development and/or maintenance of inhibitors [15]. Thus, a better understanding of how these therapies may affect immune cell profiles in children could inform both treatment decisions and broader questions related to immune development in the context of HA management. This study aims to address this gap by comparing paediatric HA patients undergoing the two different prophylaxis regimens, either Emicizumab or rFVIII. To strengthen the results, HB patients, who receive factor IX (FIX) replacement therapy and exhibit the same bleeding phenotype of HA, were included as internal controls together with healthy subjects. Eventually, we propose to investigate the immune cell compartment to assess physiological variations under these different therapeutic conditions, providing new insights into their distinct physiological impacts in early life.

## 2 Methods

### 2.1 Study Population

Paediatric patients' blood samples were obtained upon signed informed consent and were collected by our collaborators at Azienda Ospedaliera Universitaria (AOU) Città della Salute e della Scienza (Torino) and AOU SS. Antonio e Biagio e Cesare Arrigo (Alessandria). The research was approved by institutional ethical committees (Protocol n. 712/CE, Study n. ce 147/20). This study includes patients affected by HA, HB, and healthy subjects whose characteristics are listed in Table S1. Data on full blood panel, coagulation analysis and clinical biochemistry tests were obtained through medical reports at the time of sample collection. When possible, HA patients have been stratified according to their undergoing treatment at the time of sample collection, either FVIII or Emicizumab; their age, more or < 10 years; inhibitor history; by being previously untreated patients (PUPs) or previously treated patients (PTPs), < 10 infusions. PUPs were defined as patients who had never been exposed to FVIII, whereas PTPs had received limited FVIII exposure (< 10 exposure days) before switching to Emicizumab. Notably, both PUPs and PTPs belong to the Emicizumab‐treated group in this cohort. The Emicizumab‐treated subgroup is enriched in younger patients, which contributes to a lower overall mean age of the HA cohort compared to the FVIII‐treated subgroup. Moreover, HB samples were included as an internal control to assess the specificity of FVIII‐driven immune responses.

### 2.2 Sample Processing and Immune Phenotyping by Flow Cytometry

Blood samples were collected in EDTA‐tubes and processed within 24 h. First, samples were centrifuged at 1500 *g* for 10 min to obtain plasma, which was then stored in aliquots at −80°C. Whole blood was then lysed by using Red Blood Cell Lysis Buffer (RBCLB, 155 mM NH_4_Cl, 10 mM NaHCO_3_, 0.1 mM EDTA) for 10 min on ice; successively, the reaction was neutralized and centrifuged at 300 *g* for 5 min, and eventually resuspended in RPMI‐1640 (Gibco) containing 10% FBS, 1% glutamine (Sigma‐Aldrich), and 1% penicillin/streptomycin (Sigma‐Aldrich) (complete media). Cell viability was assessed by Trypan blue, and it was generally ≥ 85%. The obtained single cell suspension was stained with fluorochrome‐conjugated antibodies, listed in Table S2, and resuspended in FACS Buffer (PBS containing 0.5% BSA and 2 mM EDTA). Percentage, number/L, and activation status of blood immune populations were assessed by acquiring the samples on the Attune NxT Acoustic Focusing Cytometer (Thermofisher Scientific), and data were analysed using the FlowJo software (BD Biosciences). The number/L of the analysed cell populations was derived by multiplying the percentages obtained by flow cytometry with the corresponding white blood cell (WBC) counts, as determined from the total blood count performed on the day of sample collection for each patient. A schematic representation of the gating strategy used for evaluating all the immune populations in the whole blood sample is shown in Figure S1.

### 2.3 Milliplex Human Cytokine/Chemokine/Growth‐Factor Panel on Plasmas

The evaluation of a 48 cytokines and chemokines panel was performed on plasma samples using a magnetic bead‐based multiplex immunoassays (Human Cytokine/Chemokine/Growth‐Factor Panel A 48 Plex Kit, Cat # HCYTA‐60 K‐PX48) following manufacturers' instructions (Merck). For the standard curve, reconstituted standards were diluted in the diluent provided in the kit. 24 HA, 8 HB and 5 healthy plasma samples were analysed in duplicate. Acquisition was performed using the Bio‐Plex 200 reader (BIO‐RAD), providing information regarding the concentration (pg/mL).

### 2.4 Human Macrophage Differentiation and FVIII Stimulation

Peripheral blood cells were used to in vitro differentiate patient‐derived macrophages [16]. After lysis, whole blood cells were plated at a concentration of 1–1.5 × 10^6^ cells/mL in serum‐free RPMI‐1640. Cells were incubated at 37°C 5% CO_2_ and monocytes were allowed to adhere for 60–90 min. Non‐adherent cells were gently removed by performing few washings using serum‐free medium. Enriched monocytes were cultured in differentiation medium: RPMI 1640 10% FBS + rh‐M‐CSF (10 μg/mL) (ImmunoTools GmbH). Medium was changed every 2–3 days, and the differentiation process took around 7 to 10 days. Differentiated cells were stimulated with recombinant human FVIII (Octocog Alpha, Bayer; 1 U/mL) while non‐treated cells served as negative control. 24‐h following stimulation, supernatants and lysed cells were collected and stored at −80°C.

### 2.5 Enzyme‐Linked Immunosorbent Assay (ELISA)

ELISA assay for the detection of human cytokines MIP‐1β (CCL4), IL‐18, TNF‐α and IL‐12p40 were performed following manufacturer's instructions (Thermofisher Scientific). Cell supernatants of healthy individuals and hemophilic patient‐derived macrophages stimulated with FVIII or non‐stimulated were analysed in duplicates. Data are shown as concentrations expressed in pg/mL. The detection limits for the chemokines were: MIP‐1 (4 pg/mL), IL‐18 (6.25 pg/mL), TNF‐α (4 pg/mL) and IL‐12p40 (< 10 pg/mL). Levels below the detection limit were defined as 0 pg/mL. Due to variability in sample availability and experimental suitability (e.g., cell differentiation efficiency), not all samples were included in all analyses, resulting in differences in sample size across experiments.

### 2.6 RNA Extraction and Real‐Time qPCR

Total RNA extraction was performed using TRIzol Reagent (Invitrogen) following manufacturer's protocol. RNA samples were quantified with NanoDrop 2000 (Thermofisher Scientific) and complementary DNA (cDNA) was obtained using RevertAid H minus first Strand cDNA Synthesis Kit (Thermofisher Scientific). The obtained cDNAs were used to study the gene expression profile: primers were designed exploiting the Primer designing tool (NCBI–NIH; https://www.ncbi.nlm.nih.gov/tools/primer‐blast/) and are listed in Table S3. Real‐Time qPCR was performed with the Titan HotTaq EvaGreen qPCR Mix (ROX) (Bioatlas) and using the StepOnePlus instrument (Applied Biosystems). Data are analysed with the 2^−ΔΔCt^ method and expressed as fold change (FC) between FVIII treatment over non‐treated. Due to variability in sample availability and experimental suitability (e.g., cell differentiation efficiency), not all samples were included in all analyses, resulting in differences in sample size across experiments.

### 2.7 Statistical Analysis

For statistical analyses and charts we used GraphPad Prism 8.0 (GraphPad). Percentages of the different blood immune populations were assessed through the FlowJo software (BD Biosciences). All data are expressed as mean ± Standard Deviation (SD). Comparison among different groups was carried out with unpaired *t*‐test or Mann–Whitney test, when two groups were compared, while one‐way analysis of variance (ANOVA) has been applied when the comparison included three groups. Two‐way ANOVA was used to analyse the gene expression and cytokine analysis on patient‐derived macrophages. A *p*‐value of < 0.05 was statistically significant and the significance was expressed as follows: *p < 0.05; **p < 0.01; ***p < 0.001; ****p < 0.0001.

## 3 Results

### 3.1 Medical Reports and Circulating Immune Population Analysis

**FIGURE 1:**
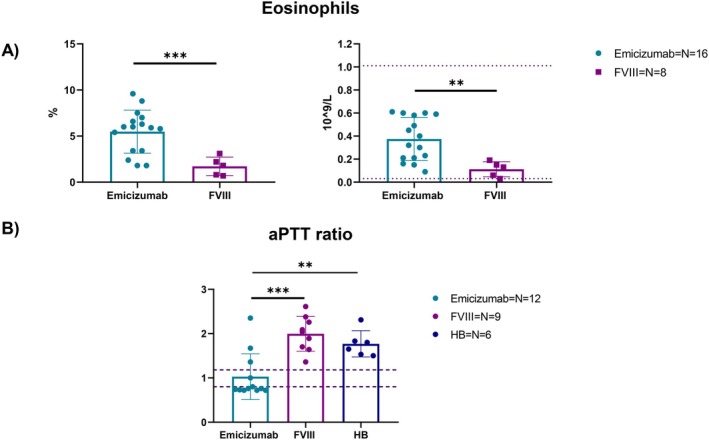
Principal findings of hospital medical reports: A comparison between Haemophilia A (HA) prophylaxis treatments and Haemophilia B (HB) in total blood count. (A) Difference in percentage and number of circulating eosinophils between FVIII‐treated and Emicizumab‐treated HA patients. (B) Comparison of aPTT ratio (ratio between the patient's clotting time and control normal pooled plasma clotting time) between all prophylactic treatments, FVIII and Emicizumab for HA and FIX for HB. All data are represented as a scatter plot where the values for individual patients are represented by dots, while the bars represent the means of the individual groups ± standard deviation (SD). The upper and lower dashed lines indicate the normal range (not pathological) values. One‐way ANOVA has been used for comparing the three groups, while unpaired *t*‐test was used when two groups were compared (*p < 0.05; **p < 0.01; ***p < 0.001; ****p < 0.0001).

**FIGURE 2:**
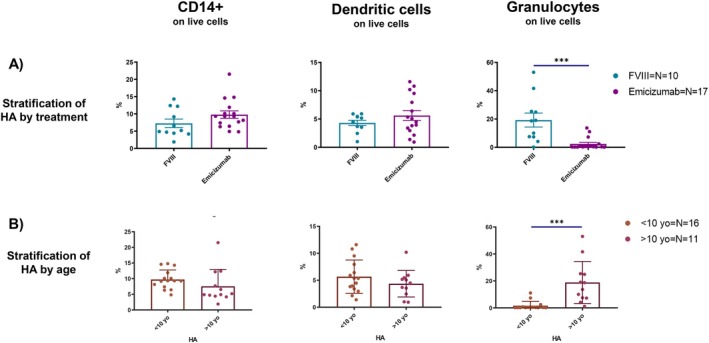
Evaluation of blood immune populations between groups. Graphs show percentages of CD14+ cells, including monocytes, dendritic cells (CD11c/SSClow) and granulocytes (CD11c/SSChigh) in peripheral blood of HA patients. (A) Values following stratification of HA patients according to their undergoing treatment, either rFVIII or Emicizumab. (B) Stratification of HA patients according to their age at the time of blood collection. All data are represented as a scatter plot where the values for individual patients are represented by dots, while the bars represent the means of the individual groups ± SD. Unpaired *t*‐test was used to compare the two groups (*p < 0.05; **p < 0.01; ***p < 0.001; ****p < 0.0001).

**FIGURE 3:**
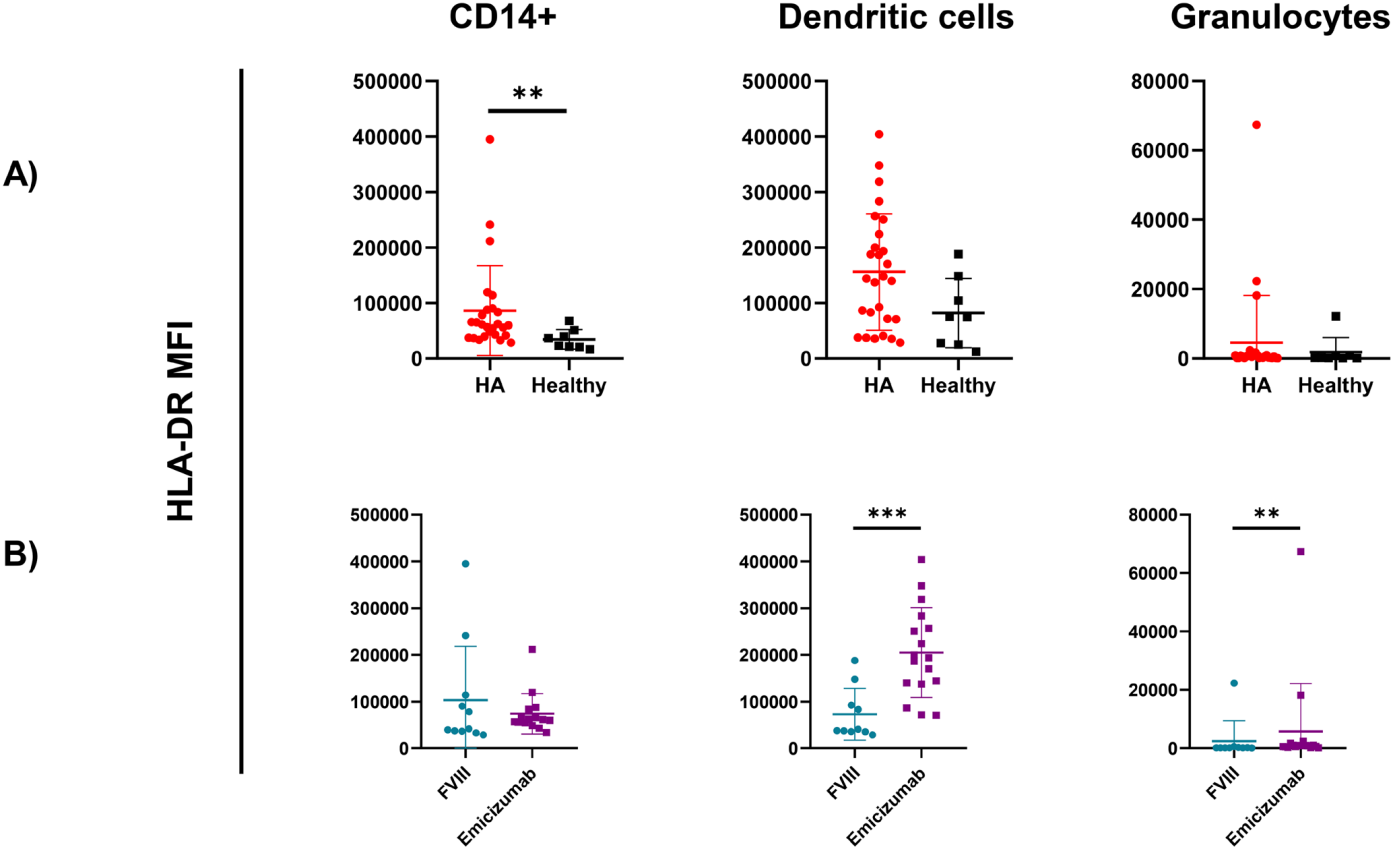
Quantification of HLA‐DR expression on blood immune cell surface through Median Fluorescent Intensity (MFI). (A) Comparison between HA and healthy CD14+ cells, dendritic cells and granulocytes. (B) Stratification of HA patients according to their undergoing treatment, either rFVIII or Emicizumab. All data are represented as a scatter plot where the values for individual patients are represented by dots, while the middle line represent the means of the individual groups ± SD. Mann–Whitney test has been used for comparing the two groups. (*p < 0.05; **p < 0.01; ***p < 0.005; ****p < 0.0001).

Our initial objective was to assess potential differences, if any, in total blood count, coagulation, and clinical biochemistry parameters between HA and HB paediatric patients; a second analysis was conducted comparing FVIII‐ versus (vs) Emicizumab‐treated HA subjects. All the parameters analysed are listed in Table 1. The number of components per group may differ since not all patients underwent the same tests at the time of sample collection. In general, it is important to underline that the values for which statistical differences are observed always fall within the normal ranges set by the paediatric medical reports and do not indicate a pathological difference. As far as total blood count is concerned, values regarding RBCs morphology and activity, such as HGB, MCV and MCH, were significantly higher in FVIII‐treated patients compared to Emicizumab‐treated. Moreover, both the percentage and number of eosinophils were significantly higher in Emicizumab‐treated patients, as shown in Figure 1A. In both cases, this difference might be associated with different mean age among the two groups [17]. Interestingly, patients in Emicizumab prophylaxis showed a lower aPTT ratio compared to both FVIII‐treated patients and HB patients undergoing FIX prophylaxis (Figure 1B). CD3+, CD14+, dendritic cells (CD11c+/SSC_low_) and granulocytes (CD11c+/SSC_hi_), CD34+ and B cells, representing both the lymphoid and myeloid compartments, were analysed in percentage and count by flow cytometry and are reported in Figures S2 and S3. CD11c + cells were divided in SSC_low_ and SSC_hi_ to better discriminate respectively dendritic cells and granulocytes, according to cell complexity. Overall, patients' cell characterization showed that FVIII‐treated patients had more circulating granulocytes than Emicizumab ones, both in percentage (Figure 2A) and number (Figure S3). Most younger patients (< 10 years old) are on Emicizumab prophylaxis; older patients (> 10 years old) are getting FVIII infusions (Figure 2B), thus this data is directly proportional to patients' age. A more in‐depth analysis was conducted to assess any variations in the HLA‐DR expression on the surface of myeloid cells. Specifically, there were higher levels of HLA‐DR on CD14+ cells in HA patients than in healthy people, in terms of median fluorescence intensity (MFI) (Figure 3A). The statistical difference was absent when HA samples were stratified according to undergoing treatment (Figure 3B and Figure S4). On the other hand, when evaluating HLA‐DR expression on granulocytes and dendritic cells in both groups, Emicizumab‐treated patients showed a higher expression compared to FVIII‐treated ones in MFI (Figure 3B).

**TABLE 1:** Summary of information from blood samples of HA and HB patient medical reports.

Total blood count						
Parameter mean (SD)	HA (*n* = 24)	HB (*n* = 7)	*p*	FVIII (*n* = 8)	Emicizumab (*n* = 16)	*p*
WBC, 10^9^/L	6.76 (1.80)	7.18 (2.22)	0.61	6.55 (1.54)	6.70 (1.95)	0.69
RBC (10^12^/L)	5.02 (0.41)	5.05 (0.48)	0.77	5.19 (0.46)	4.92 (0.36)	0.10
HGB (g/dL)	13.13 (1.70)	13.24 (1.33)	0.88	14.65 (1.60)	12.32 (1.17)	**0.0006**
HCT (%)	39.43 (4.91)	40.51 (2.37)	0.58	44.23 (4.02)	37.03 (3.32)	**0.0001**
MCV (fL)	78.8 (8.62)	80.7 (6.7)	0.59	85.4 (3.54)	75.4 (8.59)	**0.006**
MCH (pg)	26.3 (3)	26.4 (3.11)	0.94	28.2 (1.08)	25.2 (3.21)	**0.022**
MCHC (g/dL)	33.33 (1.48)	32.67 (2.06)	0.35	33.13 (1.17)	33.3 (1.64)	0.64
RDW‐CV (%)	13.92 (2.9)	13.82 (1.25)	0.70	12.99 (0.74)	13.59 (3.53)	0.64
PLTS (10^9^/L)	286.17 (120.54)	360.43 (154.71)	0.19	244.75 (92.68)	304.67 (130)	0.24
MPV (fL)	8.7 (1.4)	8.5 (1.4)	0.66	9.6 (1.1)	8.4 (1.4)	**0.021**

Differential leucocyte count						
Parameter value (SD)	HA (*n* = 24)	HB (*n* = 7)	*p*	FVIII (*n* = 8)	Emicizumab (*n* = 16)	*p*
Neutrophils (%)	47.13 (11.58)	45.14 (12.73)	0.70	54.95 (12.57)	44.29 (9.11)	**0.016**
Lymphocytes (%)	39.32 (2.24)	41.99 (13.76)	0.58	34.41 (11.79)	44.29 (8.95)	0.10
Monocytes (%)	6.63 (2.24)	7.38 (1.4)	0.40	6.05 (1.39)	6.97 (2.55)	0.38
Eosinophils (%)	4.25 (2.64)	2.8 (1.74)	0.18	1.81 (0.97)	5.62 (2.34)	**0.0004**
Basophils (%)	0.73 (0.32)	0.56 (0.15)	0.23	0.86 (0.38)	0.63 (0.28)	0.11
Neutrophils (10^9^/L)	3.21 (1.36)	3.29 (1.43)	0.89	3.74 (1.59)	2.97 (1.19)	0.17
Lymphocytes (10^9^/L)	2.6 (0.95)	2.97 (1.34)	0.42	2.12 (0.43)	2.66 (1.05)	0.08
Monocytes (10^9^/L)	0.44 (0.21)	0.54 (0.23)	0.28	0.39 (0.10)	0.46 (0.24)	0.37
Eosinophils (10^9^/L)	0.29 (0.20)	0.20 (0.13)	0.29	0.12 (0.06)	0.38 (0.19)	**0.001**
Basophils (10^9^/L)	0.05 (0.022)	0.04 (0.007)	0.31	0.06 (0.03)	0.04 (0.017)	0.13

Coagulation						
Parameter value (SD)	HA (*n* = 22)	HB (*n* = 9)	*p*	FVIII (*n* = 8)	Emicizumab (*n* = 14)	*p*
Chromogenic missing factor (%)	4.97 (7.67)	6.89 (2.37)	0.47	8.53 (9.36)	3.16 (0.33)	0.12
PT‐ratio	1.06 (0.06)	1.17 (0.09)	**0.004**	1.03 (0.05)	1.09 (0.06)	0.28
aPTT ratio	1.44 (0.67)	1.77 (0.29)	0.26	2.00 (0.39)	1.05 (0.51)	**0.0002**
p‐fibrinogen (mg/dL)	254.36 (50.77)	273.2 (96.41)	0.58	255.87 (67.82)	254.8 (15.97)	0.90

Clinical biochemistry						
Parameter value (SD)	HA (*n* = 18)	HB (*n* = 6)	*p*	FVIII (*n* = 7)	Emicizumab (*n* = 11)	*p*
s‐Glucose (mg/dL)	83.22 (9.35)	84.5 (8.43)	0.77	84 (5)	82.72 (11.54)	0.79
s‐Creatinin (mg/dL)	0.5 (0.25)	0.46 (0.17)	0.46	0.69 (0.30)	0.40 (0.13)	**0.009**
s‐Bilirubin Tot (mg/dL)	0.49 (0.35)	0.6 (0.43)	0.53	0.7 (0.45)	0.39 (0.22)	**0.042**
s‐Bilirubin direct (mg/dL)	0.17 (0.11)	0.25 (0.19)	0.24	0.24 (0.13)	0.12 (0.04)	**0.01**
s‐Bilirubin indirect (mg/dL)	0.33 (0.28)	0.45 (0.33)	0.48	0.47 (0.30)	0.27 (0.22)	0.09
s‐AST (UI/L)	28.47 (10.69)	25.5 (4.09)	0.52	21.71 (6.18)	30.72 (10.97)	**0.031**
s‐ALT (UI/L)	19.53 (9.45)	13.83 (5.23)	0.18	21.57 (13.28)	17.27 (6.76)	0.49
s‐GGT (UI/L)	12.78 (5.62)	11.67 (3.07)	0.65	16.71 (6.87)	10.5 (2.76)	**0.013**
s‐Iron (μg/dL)	87.69 (34.66)	132.4 (76.04)	0.15	98.14 (38.13)	79.56 (31.5)	0.49
s‐Transferrin (g/L)	2.74 (0.25)	2.76 (0.34)	0.66	2.79 (0.23)	2.7 (0.27)	0.63
s‐Ferritin (μg/L)	68.47 (73.84)	58.25 (24.14)	0.79	117.15 (97.89)	34.4 (13.17)	**0.017**

*Note:* Mann–Whitney test was used for comparing two groups and significance was set at *p*‐value of *p* < 0.05. The bold values are statistically significant (*p* < 0.05).

### 3.2 Plasma Cytokines Profile

**FIGURE 4:**
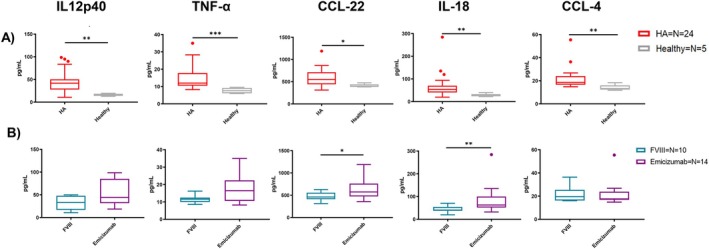
Broad spectrum of monocyte/macrophages‐related cytokines in hemophilic patient plasma. (A) Graphs showing comparison of IL12p40, TNF‐a, CCL‐22, IL‐18 and CCL‐4 quantification in plasma of HA patients and healthy donors. (B) Stratification of HA patients according to their undergoing treatment, either rFVIII or Emicizumab. All data are represented as a Tukey plot where the out‐of‐range samples are represented as a dot. Mann–Whitney test has been used for comparing the two groups (*p < 0.05; **p < 0.01; ***p < 0.001; ****p < 0.0001).

To detect changes potentially associated with a pro‐ or anti‐inflammatory state, a panel of 48 cytokines and chemokines was simultaneously analysed in plasma samples from HA, HB, and healthy individuals using a magnetic bead‐based multiplex immunoassay. This assay allows the detection of even very small concentrations of a cytokine through its coupled magnetic capture beads technology and its high sensitivity, taking advantage of fluorescent signals. The broad spectrum of the investigated cytokines is charted in Figures S5 and S6 as a log_10_ of the group's mean concentration in order to normalize and to level differences in concentration between the cytokines. Instead, the concentration of each cytokine in every group is listed in Table S4. The analysis highlighted five differently produced cytokines/chemokines among the three groups of subjects: IL‐12p40, TNF‐α, CCL‐22, IL‐18, and CCL‐4. This cytokine pattern is mainly associated with monocyte/macrophage activity, since all of them are either produced by or acting on these cells [18, 19, 20, 21]. In all cases, those cytokines were significantly more present in HA than healthy plasma (Figure 4A). However, these cytokines did not reveal a distinct immunological pattern differentiating FVIII and Emicizumab treatments since statistical analysis shows only significantly higher levels of CCL‐22 and IL‐18 in patients receiving prophylaxis with Emicizumab compared to those treated with FVIII (Figure 4B and Figure S7A).

### 3.3 Cytokine Production by Patient‐Derived Macrophages Following In Vitro FVIII Stimulation

**FIGURE 5:**
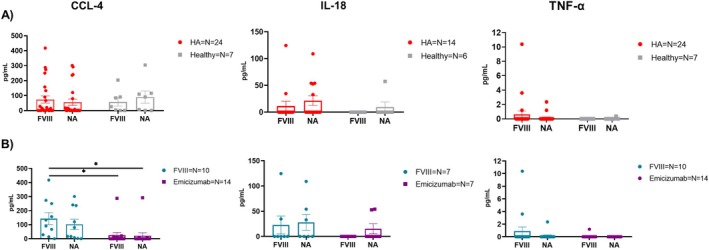
Cytokines expression from patient‐derived macrophages differentiated in vitro 24 h following FVIII stimulation. Graphs are showing CCL4, IL‐18 and TNF‐a expression from each group following FVIII stimulation (FVIII) compared to untreated (NA). (A) Comparison between HA and healthy macrophages. (B) Stratification of HA patients according to their undergoing treatment, either rFVIII or Emicizumab. All data are represented as a scatter plot where the values for individual patients are represented by dots, while the middle line represents the means of the individual groups ± SD. All undetected samples by the test were considered as 0 pg/mL. One‐way ANOVA has been used for comparing the groups (*p < 0.05; **p < 0.01; ***p < 0.001; ****p < 0.0001).

Building on above‐mentioned data, suggesting a potential role for macrophages, we decided to obtain patient‐derived macrophages to confirm whether the systemic changes observed in HA plasma were carried out by in vivo macrophages. To accomplish this, macrophages were first differentiated in vitro starting from patients' monocytes followed by an evaluation of the cytokine production in the culture supernatants. This assessment included both untreated (NA) and FVIII‐treated differentiated macrophages. The cytokines analysed were IL‐12p40, CCL‐4, IL‐18 and TNF‐α. IL‐12p40 was undetected in all samples, while no differences were observed between HA and healthy macrophages in the production of the other cytokines, despite FVIII‐stimulation (Figure 5A). However, when HA samples are stratified by prophylactic treatment, it became apparent that macrophages derived from patients undergoing FVIII prophylaxis produced higher levels of these cytokines compared to those from patients on Emicizumab prophylaxis, with a significantly higher production of CCL‐4, regardless of in vitro FVIII stimulation (Figure 5B and Figure S7B).

### 3.4 Gene Expression Pattern Identification of FVIII‐Treated Patient‐Derived Macrophages

**FIGURE 6:**
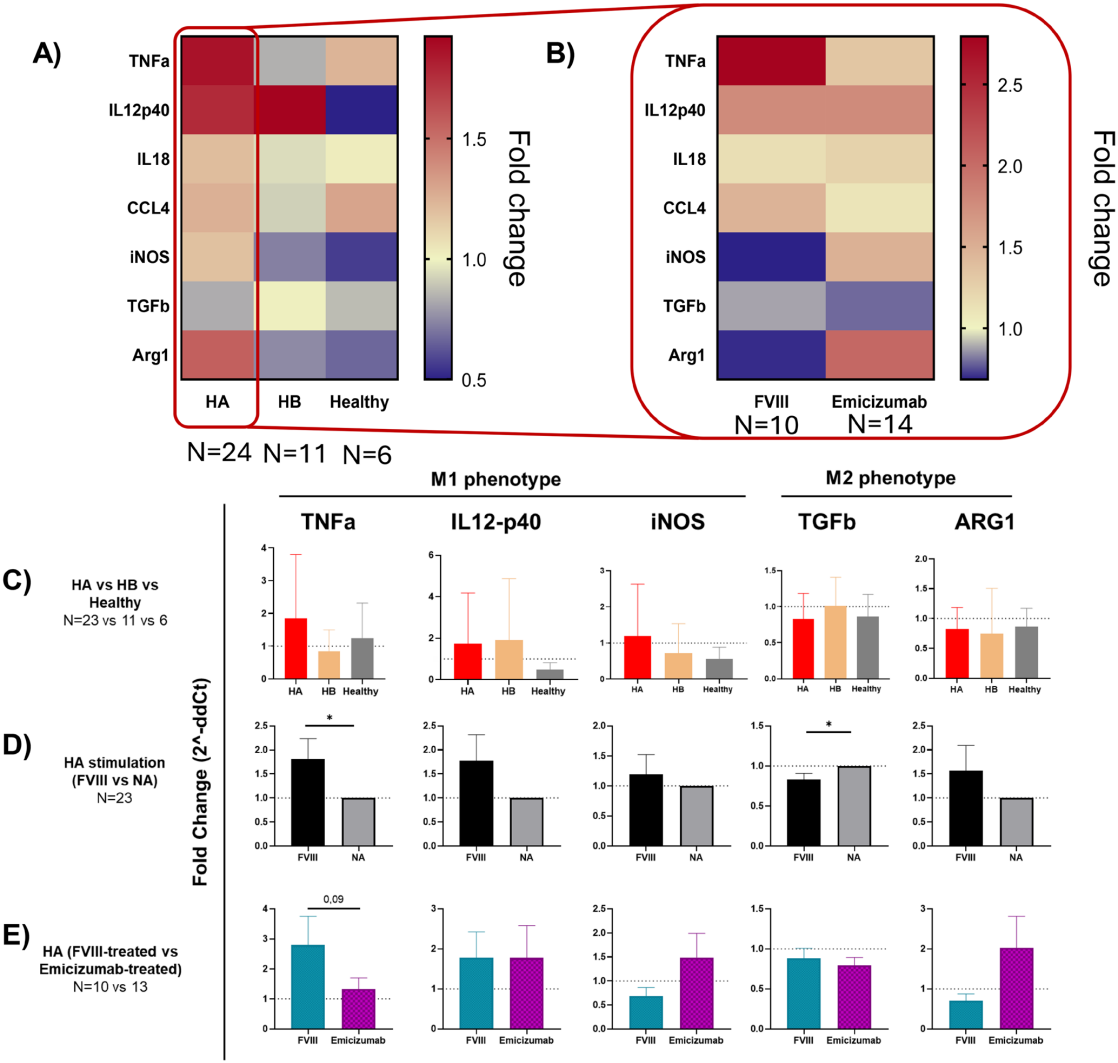
Gene expression analysis of patient‐derived macrophages stimulated with FVIII in vitro, to understand if they shift toward an M1 or M2 phenotype. (A and B) Heatmaps showing gene expression profiles of HA (*N* = 23), HB (*N* = 11) and healthy (*N* = 6) macrophages (A), and subsequent stratification of HA patients according to their undergoing treatment, either rFVIII or Emicizumab (B). Values for each gene are calculated as fold‐change of FVIII‐treated over non‐treated samples and they are represented as double‐gradient heatmap as group's mean value. Comparison of TNF‐a, IL12p40, iNOS, TGFb and ARG1 mRNAs expression between HA, HB and healthy macrophages (C), HA macrophages stimulated with FVIII and their untreated controls (D), and stratification of HA patients according to their undergoing treatment, either rFVIII or Emicizumab (E). Data are represented as histogram plots with bars representing the means of the individual groups ± SD. One‐way ANOVA or two‐way ANOVA have been used for comparing the three groups, while unpaired t‐test was used when two groups were compared (*p < 0.05; **p < 0.01; ***p < 0.001; ****p < 0.0001).

To investigate macrophages transition to a pro‐ (M1) or anti‐inflammatory (M2) phenotype in response to FVIII stimulation, we analysed the gene expression profiles of untreated and stimulated macrophages derived from HA, HB and healthy individuals. We analysed the mRNA level of *IL‐12p40*, *IL‐18*, *TNF‐α* and *CCL‐4*, as well as cytokines characterizing M1 (specifically, *TNF‐α*, *IL‐12p40* and *iNOS*) and M2 (*TGF‐β* and *Arg‐1*) phenotypes. To better dissect FVIII activity on macrophages, single values are expressed as fold‐change (FC) of FVIII‐treated over untreated samples and they are represented in a double‐gradient heatmap as group's mean value (Figure 6A). Altogether, it is possible to highlight an overall pattern similarity between HB and healthy macrophages, in which the genes are not strongly up or downregulated after FVIII administration in vitro. The only exception seems to be represented by *IL‐12p40*, where HB samples behave similarly to HA macrophages rather than those derived from healthy donors. Instead, HA macrophages appear to have a specific pattern of upregulation of most of the genes investigated (Figure 6A). Stratification of HA samples allowed to discriminate the subgroup from which the upregulation originates (Figure 6B). In particular, HA macrophages derived from patients undergoing FVIII prophylaxis have an upregulation of *TNF‐α* and a downregulation of *Arg‐1*, yet both being not statistically significant. Instead, macrophages differentiated by Emicizumab‐treated patients show a general less strong activation, mainly characterized by a higher *Arg‐1* and lower *TNF‐α* expression compared to macrophages derived from patients in prophylaxis with FVIII. To better identify the different gene expression between the groups and clarify whether there is a shift toward the M1 or M2, different analysis and stratifications have been performed on the primarily involved genes of these phenotypes (Figure 6C). As already shown by the heatmap, there are no differences in gene expression of HA, HB and healthy macrophages following FVIII administration in vitro (Figure 6C). When HA FVIII‐stimulated macrophages are compared with their corresponding untreated controls, it is possible to highlight a statistical higher expression of *TNF‐α* and a downregulation of *TGF‐β*, with a slight upregulation of *IL‐12p40* and *iNOS* as well (Figure 6D). Our results show a clear tendency of *TNF‐α* upregulation by macrophages from patients in FVIII prophylaxis and that the upregulation of *Arg‐1* is mainly occurring in macrophages derived by patients in treatment with Emicizumab (Figure 6E).

## 4 Discussion

Although the differences between various types of HA prophylaxis are well established, most published studies primarily focus on drug activity, efficacy, and ABR associated with the multiple available therapies [22, 23, 24, 25, 26, 27]. This study offers a comparison of the two common prophylactic treatments for HA, rFVIII and Emicizumab, focusing on an immunological screening of paediatric patients. For comparison, we also included paediatric HB patients given their common bleeding phenotype caused by a different coagulation factor while being unaffected by FVIII‐targeted immune responses. Medical reports concerning total blood, coagulation, and biochemistry clinical parameters highlighted that the main differences are shown once HA treatment therapies are compared. Despite our data showing higher haemoglobin levels in patients treated with rFVIII, a preliminary study has indicated that prophylaxis with Emicizumab leads to improvements in haemoglobin levels and RBC indices following prophylaxis initiation [28]. Consequently, the difference observed in our study is most likely due to the age, as patients receiving Emicizumab in our cohort were, on average, considerably younger than those treated with FVIII. On the contrary, to our knowledge, eosinophils being higher in patients under Emicizumab prophylaxis has never been reported in literature, and it could involve a combination of immune modulation and cytokine environment of the hemophilic immune system [29, 30]; however these values still fall within the normal range and could be again related to patients' age highlighting again the need to further investigate this point by increasing our cohort number and performing age‐matched analysis. In addition, the decreased aPTT ratio remarks on Emicizumab efficacy and stability, in agreement with previous literature [14, 31]. Nonetheless, it is challenging to establish a correlation between our observations and the different treatments due to the limited sample size, different age, variability in individual immune responses, and the potential influence of additional factors such as pre‐existing inflammation, genetic predispositions, or disparities in initial immune cell types.

In general, we can assert that the HA innate immune system, and specifically the myeloid compartment, shows a different capability in communicating with the adaptive immune systems, since CD14 is a key marker for the identification of human monocytes/macrophages, while CD11c is used for dendritic cells and granulocytes. This is mainly confirmed by the HLA‐DR expression on CD14+ being higher in HA cells compared to healthy ones. The difference is clear following HA patient stratification according to undergoing treatment. In fact, Emicizumab‐treated patients have decreased circulating granulocytes and higher surface expression of the HLA‐DR marker compared to FVIII‐treated patients in both granulocytes and dendritic cells. Despite age could represent a confounding factor in HLA‐DR expression, since it has been reported that it is developmentally regulated, with lower levels observed in neonates and early infancy and a progressive increase with age toward adolescence and adulthood [32, 33], our observations appear to diverge from this trend, as HA samples display higher HLA‐DR expression compared to healthy controls despite the paediatric setting. This suggests that the differences observed in our cohort are unlikely to be solely explained by age‐related variation, but rather may reflect disease‐specific immune modulation associated with haemophilia A. In fact, the higher expression on HLA‐DR on these myeloid cells suggests immune activation and an increased capability of these antigen‐presenting cells in communicating with the adaptive immune system [34, 35]. Overall, this is also confirmed by the differently modulated cytokines in HA plasma, all produced by or acting on monocytes and macrophages. In fact, monocytes and macrophages are the primary producers of TNF‐α, IL‐18, and CCL‐4: the first two are involved in INF‐γ production and, in particular, IL‐18 works in synergy with IL‐12 to stimulate INF‐γ production [36, 37]. Anyhow, all the aforementioned cytokines are produced within ranges described by literature [38, 39]. It is possible to rather describe a chemoattractant activity for cells of innate immunity, like dendritic cells, granulocytes and monocytes/macrophages [20, 21, 36]. The gene expression analyses better clarify the behaviour of HA macrophages following in vitro FVIII administration rather than their cytokine production. One explanation could be associated with the time point chosen: due to scarcity of the sample, it was possible just to investigate one time point per sample, and 24 h seemed to be a better fit for both experimental tests. Studies on cytokine production comparing HA, HB, and healthy macrophages confirm that FVIII supplementation in the culture media does not induce a specific cytokine pattern. The only distinction arises from the patients these cells were differentiated from, undergoing different prophylaxis. Moreover, our in vitro experiments showed no production of the cytokines found to be dysregulated in patients' plasma, hinting that either macrophages are not the primary source of these cytokines in vivo, or that FVIII alone is insufficient to trigger their production by macrophages in our experimental conditions.

Macrophage polarization dysfunction in HA has already been described [40, 41, 42] and this could have implications on the inflammatory milieu [43, 44]. Here as well, we show through gene expression analysis that all HA macrophages appear to switch to an M1‐like phenotype following in vitro FVIII stimulation, regardless of the prophylaxis regimen. This is primarily supported by the significant upregulation of *TNF‐α* and downregulation of *TGF‐β* when FVIII‐stimulated HA and untreated macrophages are compared. Interestingly, further stratification based on the type of prophylaxis treatment revealed distinct trends. Macrophages from patients undergoing FVIII prophylaxis showed a trend in upregulating *TNF‐α*, reinforcing the M1 phenotype association in these cells. Conversely, those from patients treated with Emicizumab displayed another trend in upregulating *Arg‐1*, a marker of M2‐like activity, indicating a potential differential impact of the prophylaxis on macrophage polarization. In general, we can describe macrophages from FVIII‐treated patients as “more prone to activation”, since they easily tend to switch to an M1‐like phenotype, while macrophages from Emicizumab‐treated patients are less strongly activated and there is not a clear shift toward M1 or M2.

In summary, our findings indicate differences in the myeloid compartment of HA patients and, possibly, distinct different interactions with the adaptive immune system between Emicizumab‐treated and FVIII‐treated patients. Our data show a higher recall or activation of HA monocytes‐macrophages in vitro and in vivo and highlight the complexity of macrophage responses in HA patients, particularly under different prophylactic treatments. Importantly, the differences observed between FVIII‐ and Emicizumab‐treated patients may have relevant translational implications. While patients in replacement therapy are continuously exposed to FVIII and may continuously engage antigen‐presenting cells, Emicizumab acts independently of FVIII and does not directly stimulate the same immune pathways. As a result, patients receiving Emicizumab may develop and maintain a distinct baseline immune profile, particularly within the myeloid compartment. Therefore, our findings suggest that the type of prophylactic treatment may not only control bleeding but also shape the immune landscape in a way that could impact long‐term immunogenicity to FVIII and inhibitor development. Ultimately, this study contributes valuable insights to paediatric HA patients, expanding the understanding necessary to optimize therapeutic strategies while minimizing or preventing adverse effects.

## Author Contributions

**Berardino Pollio:** methodology, conceptualization, writing – review and editing, resources. **Roberto Albiani:** conceptualization, writing – review and editing. **Irene Ricca:** conceptualization, methodology, writing – review and editing. **Tiziano Martini:** conceptualization, methodology, writing – review and editing, data curation. **Patrizia Sciancalepore:** conceptualization, writing – review and editing. **Saicharan Akula:** writing – review and editing, conceptualization. **Roberto Santi:** conceptualization, writing – review and editing. **Jacopo Agnelli Giacchello:** conceptualization, writing – review and editing. **Alessia Cottonaro:** investigation, writing – original draft, writing – review and editing, data curation, conceptualization, formal analysis, project administration, methodology. **Antonia Follenzi:** conceptualization, funding acquisition, writing – original draft, writing – review and editing, supervision, methodology. **Simone Merlin:** conceptualization, funding acquisition, writing – original draft, writing – review and editing, supervision, methodology.

## Funding

This work was supported by Roche per la Ricerca 2019, by Grifols “Martin Villar” Haemostasis Award 2019, by the ‘Fondo Ateneo per la Ricerca (FAR) 2017’ from the University of Piemonte Orientale to SM, and by PNRR MUR—M4C2—CN RNA & GENE THERAPY—Spoke 1 (Grant: ALLEVIATE) to AF.

## Ethics Statement

This study was approved by the institutional ethical committees (Protocol n. 712/CE, Study n. ce 147/20). All procedures were conducted in accordance with relevant guidelines and regulations.

## Consent

Written informed consent was obtained from all participants and/or their legal guardians prior to inclusion in the study.

## Conflicts of Interest

The authors declare no conflicts of interest.

## Supporting information


**Table S1:** Study population characteristics.
**Table S2:** Flow cytometry antibodies.
**Table S3:** List of oligonucleotides used as Forward and Reverse for Real‐Time qPCR.
**Table S4:** Group mean values of plasma cytokines in patients with HA, HB, and healthy controls. The last column indicates statistical comparisons between groups, with symbols indicating significant differences at *p* < 0.05: † = HA vs. HB; ‡ = HA vs. Healthy; § = HB vs. Healthy; / = no difference.
**Figure S1:** Schematic representation of gating strategy used for analysed immune cell populations. (A) Gating for CD3+, CD11c+ (hi = granulocytes; and low = dendritic cells), CD14+ and CD34+ cells. (B) The gating strategy for B cells was performed by selecting the negative events for CD14, CD11c and CD3 and then included the physical parameters. Each graph was first created on singlets, then live cells according to physical parameters and the next gates were adapted from its corresponding negative samples.
**Figure S2:** Flow cytometry evaluation of all the blood immune populations analysed expressed as % on live cells. Comparison between HAvs HB patients, FVIII‐ vs. Emicizumab‐treated HA patients and < 10 year‐ vs. > 10 year‐old HA patients. (A) Percentage of CD3+ cells. (B) Percentage of B cells. (C) Percentage of CD14+ cells. (D) Percentage of dendritic cells. (E) Percentage of granulocytes. (F) Percentage of CD34+ cells. All data are represented as scatter plot with bar where the upper part of the bar is equal to the mean of the individual values belonging to the groups and the standard deviation (SD) is calculated. One‐way ANOVA has been used for comparing the three groups, while unpaired *t*‐test was used when two groups were compared (*p < 0.05; **p < 0.01; ***p < 0.001; ****p < 0.0001).
**Figure S3:** Flow cytometry evaluation of all the blood immune populations analysed expressed as number on live cells. Comparison between HA vs. HB patients, FVIII‐ vs. Emicizumab‐treated HA patients and < 10 year‐ vs. > 10 year‐old HA patients. The number/L of the analysed cell populations were derived by multiplying the percentages obtained by flow cytometry with the corresponding white blood cell (WBC) counts, as determined from the total blood count performed on the day of sample collection for each patient. All data are represented as scatter plot with bar where the upper part of the bar is equal to the mean of the individual values belonging to the groups and the standard deviation (SD) is calculated. Unpaired t‐test was used to compare the two groups (*p < 0.05; **p < 0.01; ***p < 0.001; ****p < 0.0001).
**Figure S4:** Quantification of HLA‐DR expression on blood immune cell surface through Median Fluorescent Intensity (MFI). Stratification of HA patients according to their age (< 10 years old and > 10 years old) and undergoing treatment, either rFVIII or Emicizumab. All data are represented as a scatter plot where the values for individual patients are represented by dots, while the middle line represents the means of the individual groups ± SD. Two‐way ANOVA has been used for comparing the two groups (*p < 0.05; **p < 0.01; ***p < 0.005; ****p < 0.0001).
**Figure S5:** Plasma cytokine comparison between HA (*n* = 24), HB (*n* = 8) and healthy (*n* = 5) subjects. Values are detected as concentration (pg/mL) for each sample and they are represented as a single‐gradient heatmap as group's log_10_ (mean value).
**Figure S6:** Plasma cytokine comparison between HA patients in FVIII (*n* = 8) or Emicizumab (*n* = 15) prophylaxis. Values are detected as concentration (pg/mL) for each sample and they are represented as a single‐gradient heatmap as group's log10 (mean value).
**Figure S7:** Monocyte/macrophages‐related cytokines. (A) Graphs showing comparison of IL12p40, TNF‐a, CCL‐22, IL‐18 and CCL‐4 quantification in plasma of HA patients stratified according to their undergoing treatment, either rFVIII or Emicizumab, and healthy donors. (B) Graphs are showing CCL4, IL‐18 and TNF‐a expression by monocyte‐derived macrophages from each group following FVIII stimulation (FVIII) compared to untreated (NA). Comparison between HA macrophages, stratified according to their undergoing treatment, either rFVIII or Emicizumab, and healthy macrophages. All data are represented as a scatter plot where the values for individual patients are represented by dots, while the middle line represents the means of the individual groups ± SD. All undetected samples by the test were considered as 0 pg/mL. One‐way ANOVA has been used for comparing the groups (*p < 0.05; **p < 0.01; ***p < 0.001; ****p < 0.0001).

## Data Availability

The data that support the findings of this study are available from the corresponding author upon reasonable request.

## References

[1] P. S. Nair , S. Shetty , and K. Ghosh , “Factor VIII Antigen, Activity, and Mutations in Hemophilia A,” Clinical and Applied Thrombosis/Hemostasis 22, no. 4 (2016): 381–385, 10.1177/1076029614562951. 25550078

[2] E. Berntorp , K. Fischer , D. P. Hart , et al., “Haemophilia,” Nature Reviews. Disease Primers 7, no. 1 (2021): 45, 10.1038/s41572-021-00278-x. 34168126

[3] G. Castaman and D. Matino , “Hemophilia A and B: Molecular and Clinical Similarities and Differences,” Haematologica 104, no. 9 (2019): 1702–1709, 10.3324/haematol.2019.221093. 31399527 PMC6717582

[4] F. Peyvandi , I. Garagiola , and G. Young , “The Past and Future of Haemophilia: Diagnosis, Treatments, and Its Complications,” Lancet 388, no. 10040 (2016): 187–197, 10.1016/S0140-6736(15)01123-X. 26897598

[5] A. C. Weyand and S. W. Pipe , “New Therapies for Hemophilia,” Blood 133, no. 5 (2019): 389–398, 10.1182/BLOOD-2018-08-872291. 30559264

[6] J. S. Powell , N. C. Josephson , D. Quon , et al., “Safety and Prolonged Activity of Recombinant Factor VIII fc Fusion Protein in Hemophilia A Patients,” Blood 119, no. 13 (2012): 3031–3037, 10.1182/blood-2011-09-382846. 22223821 PMC3646317

[7] P. L. Turecek , M. J. Bossard , M. Graninger , et al., “BAX 855, a PEGylated rFVIII Product With Prolonged Half‐Life: Development, Functional and Structural Characterisation,” Hämostaseologie 32, no. 1 (2012): S29–S38, 10.1055/s-0037-1619772. 22961422

[8] C. Nakar and A. Shapiro , “Hemophilia A With Inhibitor: Immune Tolerance Induction (ITI) in the Mirror of Time,” Transfusion and Apheresis Science 58, no. 5 (2019): 578–589, 10.1016/j.transci.2019.08.008. 31447396

[9] K. Fischer , R. Lassila , F. Peyvandi , et al., “Inhibitor Development According to Concentrate After 50 Exposure Days in Severe Hemophilia: Data From the European HAemophilia Safety Surveillance (EUHASS),” Research and Practice in Thrombosis and Haemostasis 8, no. 4 (2024): 102461, 10.1016/j.rpth.2024.102461. 39026659 PMC11255940

[10] S. Merlin and A. Follenzi , “Escape or Fight: Inhibitors in Hemophilia A,” Frontiers in Immunology 11 (2020): 476, 10.3389/fimmu.2020.00476. 32265927 PMC7105606

[11] E. Berntorp , C. Hermans , A. Solms , L. Poulsen , and M. E. Mancuso , “Optimising Prophylaxis in Haemophilia A: The Ups and Downs of Treatment,” Blood Reviews 50 (2021): 100852, 10.1016/j.blre.2021.100852. 34243987

[12] P. E. A. Andrade , P. M. Manucci , and C. M. Kessler , “Emicizumab: The Hemophilia A Game Changer,” Haematologica 20 (2023): 12–23, 10.3324/haematol.2022.282099. PMC11063855 37916312

[13] M. Shima , K. Nogami , S. Nagami , et al., “A Multicentre, Open‐Label Study of Emicizumab Given Every 2 or 4 Weeks in Children With Severe Haemophilia A Without Inhibitors,” Haemophilia 25, no. 6 (2019): 979–987, 10.1111/hae.13848. 31515851 PMC6900083

[14] S. W. Pipe , P. Collins , C. Dhalluin , et al., “Emicizumab Prophylaxis in Infants With Hemophilia A (HAVEN 7): Primary Analysis of a Phase 3b Open‐Label Trial,” Blood 143, no. 14 (2024): 1355–1364, 10.1182/blood.2023021832. 38127586 PMC11033591

[15] A. F. Karim , A. R. Soltis , N. P. Ewing , et al., “Hemophilia A Inhibitor Subjects Show Unique PBMC Gene Expression Profiles That Include Up‐Regulated Innate Immune Modulators,” Frontiers in Immunology 11 (2020): 1219, 10.3389/fimmu.2020.01219. 32595650 PMC7303277

[16] D. Zanolini , S. Merlin , M. Feola , et al., “Extrahepatic Sources of Factor VIII Potentially Contribute to the Coagulation Cascade Correcting the Bleeding Phenotype of Mice With Hemophilia A,” Haematologica 100, no. 7 (2015): 881–892, 10.3324/haematol.2014.123117. 25911555 PMC4486222

[17] E.‐H. Nah , S. Kim , S. Cho , and H.‐I. Cho , “Complete Blood Count Reference Intervals and Patterns of Changes Across Pediatric, Adult, and Geriatric Ages in Korea,” Annals of Laboratory Medicine 38, no. 6 (2018): 503–511, 10.3343/alm.2018.38.6.503. 30027692 PMC6056383

[18] C. Atri , F. Z. Guerfali , and D. Laouini , “Role of Human Macrophage Polarization in Inflammation During Infectious Diseases,” International Journal of Molecular Sciences 19, no. 6 (2018): 1801, 10.3390/ijms19061801. 29921749 PMC6032107

[19] A. M. Cooper and S. A. Khader , “IL‐12p40: An Inherently Agonistic Cytokine,” Trends in Immunology 28, no. 1 (2007): 33–38, 10.1016/j.it.2006.11.002. 17126601

[20] Y. Y. Yazıcı , S. Belkaya , and E. Timucin , “A Small Non‐Interface Surface Epitope in Human IL18 Mediates the Dynamics and Self‐Assembly of IL18‐IL18BP Heterodimers,” Computational and Structural Biotechnology Journal 21 (2023): 3522–3531, 10.1016/j.csbj.2023.06.021. 37484491 PMC10362265

[21] Z. R. Korobova , N. A. Arsentieva , and A. A. Totolian , “Macrophage‐Derived Chemokine MDC/CCL22: An Ambiguous Finding in COVID‐19,” International Journal of Molecular Sciences 24, no. 17 (2023): 13083, 10.3390/ijms241713083. 37685890 PMC10487728

[22] S. W. Pipe , M. Shima , M. Lehle , et al., “Efficacy, Safety, and Pharmacokinetics of Emicizumab Prophylaxis Given Every 4 Weeks in People With Haemophilia A (HAVEN 4): A Multicentre, Open‐Label, Non‐Randomised Phase 3 Study,” Lancet Haematology 6, no. 6 (2019): e295–e305, 10.1016/S2352-3026(19)30054-7. 31003963

[23] L. A. Valentino , V. Mamonov , A. Hellmann , et al., “A Randomized Comparison of Two Prophylaxis Regimens and a Paired Comparison of On‐Demand and Prophylaxis Treatments in Hemophilia A Management,” Journal of Thrombosis and Haemostasis 10, no. 3 (2012): 359–367, 10.1111/j.1538-7836.2011.04611.x. 22212248 PMC3488301

[24] G. Young , J. Mahlangu , R. Kulkarni , et al., “Recombinant Factor VIII fc Fusion Protein for the Prevention and Treatment of Bleeding in Children With Severe Hemophilia A,” Journal of Thrombosis and Haemostasis 13, no. 6 (2015): 967–977, 10.1111/jth.12911. 25912075

[25] A. Tiede , F. A. Karim , V. Jiménez‐Yuste , et al., “Factor VIII Activity and Bleeding Risk During Prophylaxis for Severe Hemophilia A: A Population Pharmacokinetic Model,” Haematologica 106, no. 7 (2020): 1902–1909, 10.3324/haematol.2019.241554. PMC8252934 32327501

[26] L. Malec , F. Peyvandi , A. K. C. Chan , et al., “Efanesoctocog Alfa Prophylaxis for Children With Severe Hemophilia A,” New England Journal of Medicine 391, no. 3 (2024): 235–246, 10.1056/NEJMoa2312611. 39018533

[27] J. Mahlangu , V. Jiménez‐Yuste , G. Ventriglia , et al., “Long‐Term Outcomes With Emicizumab in Hemophilia A Without Inhibitors: Results From the HAVEN 3 and 4 Studies,” Research and Practice in Thrombosis and Haemostasis 8, no. 2 (2024): 102364, 10.1016/j.rpth.2024.102364. 38559572 PMC10978536

[28] J. Batsuli , Hemoglobin Improvement Among Patients With Hemophilia A Treated With Emicizumab Prophylaxis: Post Hoc Analysis of the HAVEN 1, 3, 4 and STASEY Trials (ISTH, 2024).

[29] F. Davoine and P. Lacy , “Eosinophil Cytokines, Chemokines, and Growth Factors: Emerging Roles in Immunity,” Frontiers in Immunology 5 (2014): 570, 10.3389/fimmu.2014.00570. 25426119 PMC4225839

[30] T. Wen and M. E. Rothenberg , “The Regulatory Function of Eosinophils,” Microbiology Spectrum 4, no. 5 (2016): 4506, 10.1128/microbiolspec.MCHD-0020-2015. PMC5088784 27780017

[31] J. I. Adamkewicz , D. C. Chen , and I. Paz‐Priel , “Effects and Interferences of Emicizumab, a Humanised Bispecific Antibody Mimicking Activated Factor VIII Cofactor Function, on Coagulation Assays,” International Journal of Laboratory Hematology 119, no. 7 (2019): 1158. 10.1055/s-0039-1688687 31064025

[32] A. Birle , C. T. Nebe , and P. Gessler , “Age‐Related Low Expression of HLA‐DR Molecules on Monocytes of Term and Preterm Newborns With and Without Signs of Infection,” Journal of Perinatology 23, no. 4 (2003): 294–299, 10.1038/sj.jp.7210906. 12774136

[33] N. P. Boeddha , D. Kerklaan , A. Dunbar , et al., “HLA‐DR Expression on Monocyte Subsets in Critically Ill Children,” Pediatric Infectious Disease Journal 37, no. 10 (2018): 1034–1040, 10.1097/INF.0000000000001990. 29570588

[34] R. Afify , K. Lipsius , S. J. Wyatt‐Johnson , and R. R. Brutkiewicz , “Myeloid Antigen‐Presenting Cells in Neurodegenerative Diseases: A Focus on Classical and Non‐Classical MHC Molecules,” Frontiers in Neuroscience 18 (2024): 1488382, 10.3389/fnins.2024.1488382. 39720231 PMC11667120

[35] M. Roerden , M. Märklin , H. R. Salih , et al., “Expression Levels of HLA‐DR in Acute Myeloid Leukemia: Implications for Antigenicity and Clinical Outcome,” Leukemia & Lymphoma 62 (2021): 659, 10.1080/10428194.2021.1885659. 33648413

[36] T. Hamza , J. B. Barnett , and B. Li , “Interleukin 12 a Key Immunoregulatory Cytokine in Infection Applications,” International Journal of Molecular Sciences 11, no. 3 (2010): 789–806, 10.3390/ijms11030789. 20479986 PMC2869233

[37] K. S. Wang , D. A. Frank , and J. Ritz , “Interleukin‐2 Enhances the Response of Natural Killer Cells to Interleukin‐12 Through Up‐Regulation of the Interleukin‐12 Receptor and STAT4,” Blood 95, no. 10 (2000): 3183–3190, 10.1182/blood.V95.10.3183. 10807786

[38] M.‐L. Decker , V. Gotta , S. Wellmann , and N. Ritz , “Cytokine Profiling in Healthy Children Shows Association of Age With Cytokine Concentrations,” Scientific Reports 7, no. 1 (2017): 17842, 10.1038/s41598-017-17865-2. 29259216 PMC5736560

[39] G. Kleiner , A. Marcuzzi , V. Zanin , L. Monasta , and E. G. Zauli , “Cytokine Levels in the Serum of Healthy Subjects,” Mediators of Inflammation 2013 (2013): 1–6, 10.1155/2013/434010. PMC3606775 23533306

[40] L. Knowles , D. Kagiri , M. Bernard , E. Schwarz , H. Eichler , and J. Pilch , “Macrophage Polarization Is Deregulated in Haemophilia,” Thrombosis and Haemostasis 119, no. 2 (2019): 234–245, 10.1055/s-0038-1676796. 30650445

[41] K. Kis‐Toth , G. M. Rajani , A. Simpson , et al., “Recombinant Factor VIII fc Fusion Protein Drives Regulatory Macrophage Polarization,” Blood Advances 2, no. 21 (2018): 2904–2916, 10.1182/bloodadvances.2018024497. 30396910 PMC6234359

[42] N. Pang , M. Ding , H. Yang , et al., “Iron Overload Causes Macrophages to Produce a Pro‐Inflammatory Phenotype in the Synovium of Hemophiliac Arthritis via the Acetyl‐p53 Pathway,” Haemophilia 30, no. 1 (2024): 195–203, 10.1111/hae.14905. 38058260

[43] G. Arango Duque and A. Descoteaux , “Macrophage Cytokines: Involvement in Immunity and Infectious Diseases,” Frontiers in Immunology 5 (2014): 491, 10.3389/fimmu.2014.00491. 25339958 PMC4188125

[44] D. M. Mosser and J. P. Edwards , “Exploring the Full Spectrum of Macrophage Activation,” Nature Reviews Immunology 8, no. 12 (2008): 958–969, 10.1038/nri2448. PMC2724991 19029990

