# Supplementary material for: Myeloid Cell Function and Cytokine Profiles in Paediatric Haemophilia A: Insights From FVIII and Emicizumab Prophylaxis
Source: J Cell Mol Med. 2026 May 27;30(11):e71216. doi: 10.1111/jcmm.71216 (PMC13240037; doi:10.1111/jcmm.71216)
Supplement: Supplementary file 1 — Table S1: Study population characteristics. Table S2: Flow cytometry antibodies. Table S3: List of oligonucleotides used as Forward and Reverse for Real‐Time qPCR. Table S4: Group mean values of plasma cytokines in patients with HA, HB, and healthy controls. The last column indicates statistical comparisons between groups, with symbols indicating significant differences at p < 0.05: † = HA vs. HB; ‡ = HA vs. Healthy; § = HB vs. Healthy; / = no difference. Figure S1: Schematic representation of gating strategy used for analysed immune cell populations. (A) Gating for CD3+, CD11c+ (hi = granulocytes; and low = dendritic cells), CD14+ and CD34+ cells. (B) The gating strategy for B cells was performed by selecting the negative events for CD14, CD11c and CD3 and then included the physical parameters. Each graph was first created on singlets, then live cells according to physical parameters and the next gates were adapted from its corresponding negative samples. Figure S2: Flow cytometry evaluation of all the blood immune populations analysed expressed as % on live cells. Comparison between HAvs HB patients, FVIII‐ vs. Emicizumab‐treated HA patients and < 10 year‐ vs. > 10 year‐old HA patients. (A) Percentage of CD3+ cells. (B) Percentage of B cells. (C) Percentage of CD14+ cells. (D) Percentage of dendritic cells. (E) Percentage of granulocytes. (F) Percentage of CD34+ cells. All data are represented as scatter plot with bar where the upper part of the bar is equal to the mean of the individual values belonging to the groups and the standard deviation (SD) is calculated. One‐way ANOVA has been used for comparing the three groups, while unpaired t‐test was used when two groups were compared (*p < 0.05; **p < 0.01; ***p < 0.001; ****p < 0.0001). Figure S3: Flow cytometry evaluation of all the blood immune populations analysed expressed as number on live cells. Comparison between HA vs. HB patients, FVIII‐ vs. Emicizumab‐treated HA patients and < 10 year‐ vs. > 10 year‐old [file JCMM-30-e71216-s001.docx]

**Supplementary data**

**Table S1. Study population characteristics.**

|  |  |
| --- | --- |
| Groups, n (%)  HA  HB  Healthy | 47 (100)  28 (59,57)  11 (23,40)  8 (17,3) |
| Age at time of sample collection, mean (SD)  HA  HB  Healthy | 10,28 (7,97)  9,36 (9,29)  27,37 (4,74) |
| Treatment status of HA patients, n (%)  rFVIII  Emicizumab | 28 (100)  10 (35,71)  18 (64,29) |
| Age of HA treated patients, mean (SD)  rFVIII  Emicizumab | 17,8 (8,05)  6,11 (3,84) |
| Inhibitor history in HA, n (%)  On going  Tolerized | 5 (17,86)  3/5 (60)  2/5 (40) |
| Previously untreated patients (PUPs), n (%) | 3/28 (10,71) |
| Previously treated patients (PTPs), n (%) | 3/28 (10,71) |
| HA severity, n (%) | 28 severe (100) |
| HA genotype, n (%)  Unknown  Intron 22 inversion  Missense mutation | 28 (100)  16 (57,1)  7 (25)  5 (17,9) |

**Table S2. Flow cytometry antibodies.**

| Antibody | Clone | Brand | Dilution |
| --- | --- | --- | --- |
| *Anti-Human CD34 PE* | 4H11[APG] | eBioscience | 1:50 |
| *Anti-Human CD33 AF700* | WM53 | eBioscience | 1:50 |
| *Anti-Human CD3 Pe-Cy5* | UCHT1 | eBioscience | 1:25 |
| *Anti-Human CD11c PE* | 3.9 | eBioscience | 1:25 |
| *Anti-Human CD14 AF780* | 61D3 | eBioscience | 1:50 |
| *Anti-Human HLA-DR Pe-Cy7* | LN3 | eBioscience | 1:75 |

**
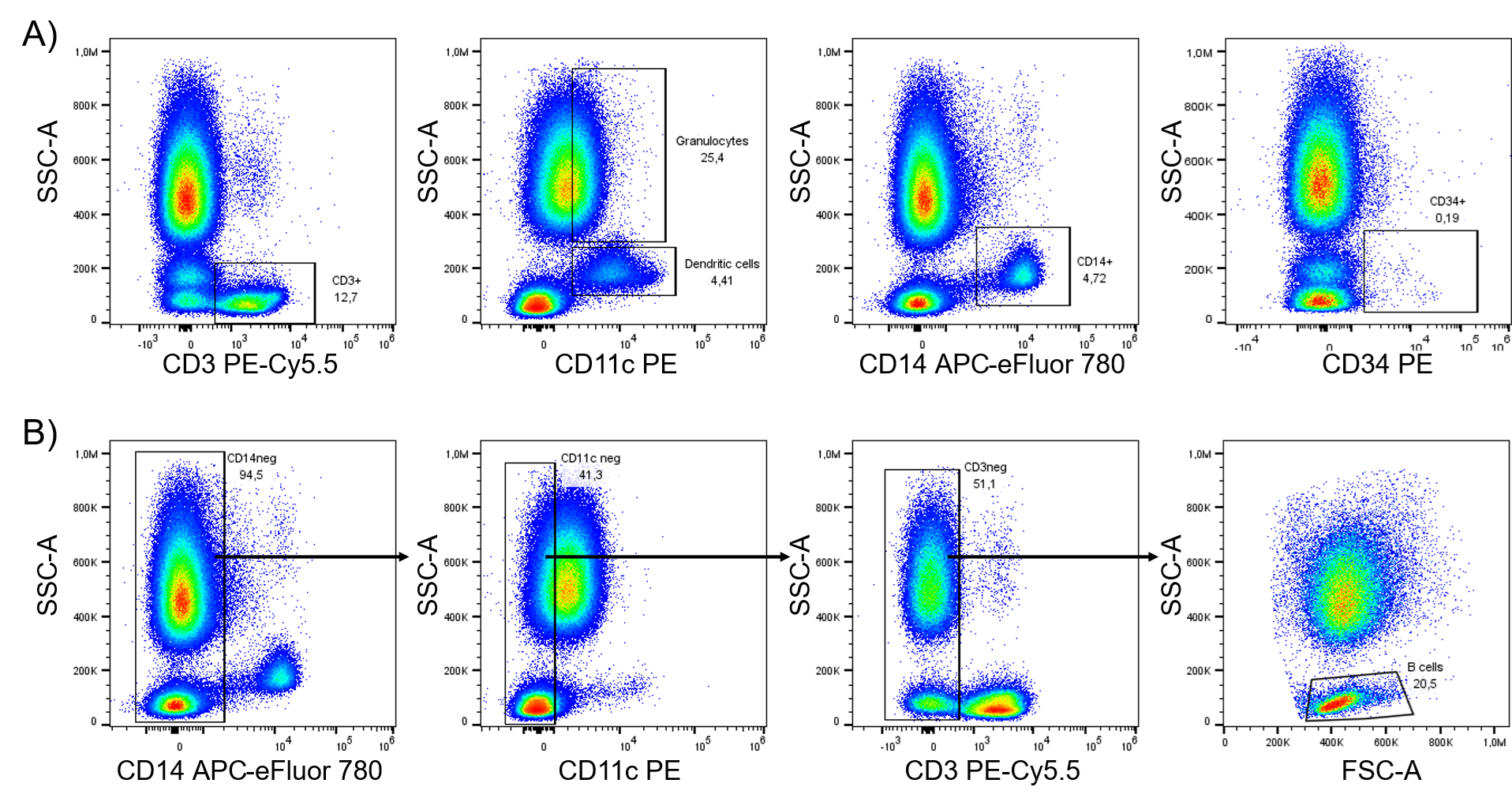
**

**Figure S1. Schematic representation of gating strategy used for analyzed immune cell populations.** A) Gating for CD3+, CD11c+(hi = granulocytes; and low = dendritic cells), CD14+ and CD34+ cells. B) The gating strategy for B cells was performed by selecting the negative events for CD14, CD11c and CD3 and then included the physical parameters. Each graph was first created on singlets, then live cells according to physical parameters and the next gates were adapted from its corresponding negative samples.

**Table S3. List of oligonucleotides used as Forward and Reverse for Real-Time qPCR.**

| **PRIMER NAME** | **PRIMER SEQUENCE** | **AMPLICON SIZE** |
| --- | --- | --- |
| *hTNF-α* | **F** CACAGTGAAGTGCTGGCAAC  **R** AGGAAGGCCTAAGGTCCACT | 180 bp |
| *hIL12p40* | **F** GCGGAGCTGCTACACTCTC  **R** CCATGACCTCAATGGGCAGAC | 126 bp |
| *hIL-18* | **F** TCTTCATTGACCAAGGAAATCGG  **R** TCCGGGGTGCATTATCTCTAC | 75 bp |
| *hCCL4* | **F** CTGTGCTGATCCCAGTGAATC  **R** TCAGTTCAGTTCCAGGTCATACA | 61 bp |
| *hiNOS* | **F** TGCGAGTTCCCCTTCTAGCG  **R** CCCGCCCCTTCAAGGAAAGA | 240 bp |
| *hTGF-β* | **F** CCAGGAACCCTTTCGCCCACCAAAC  **R** TGCTGGTTGCTGGTTTGGAGGAA | 113 bp |
| *hARG1* | **F** ACTTAAAGAACAAGAGTGTGATGTG  **R** CATGGCCAGAGATGCTTCCA | 218 bp |
| *hβ-actin* | **F** TGCCTGACGGCCAGGTCAT  **R** ATCTCCTTCTGCATCCTGTCGG | 226 bp |


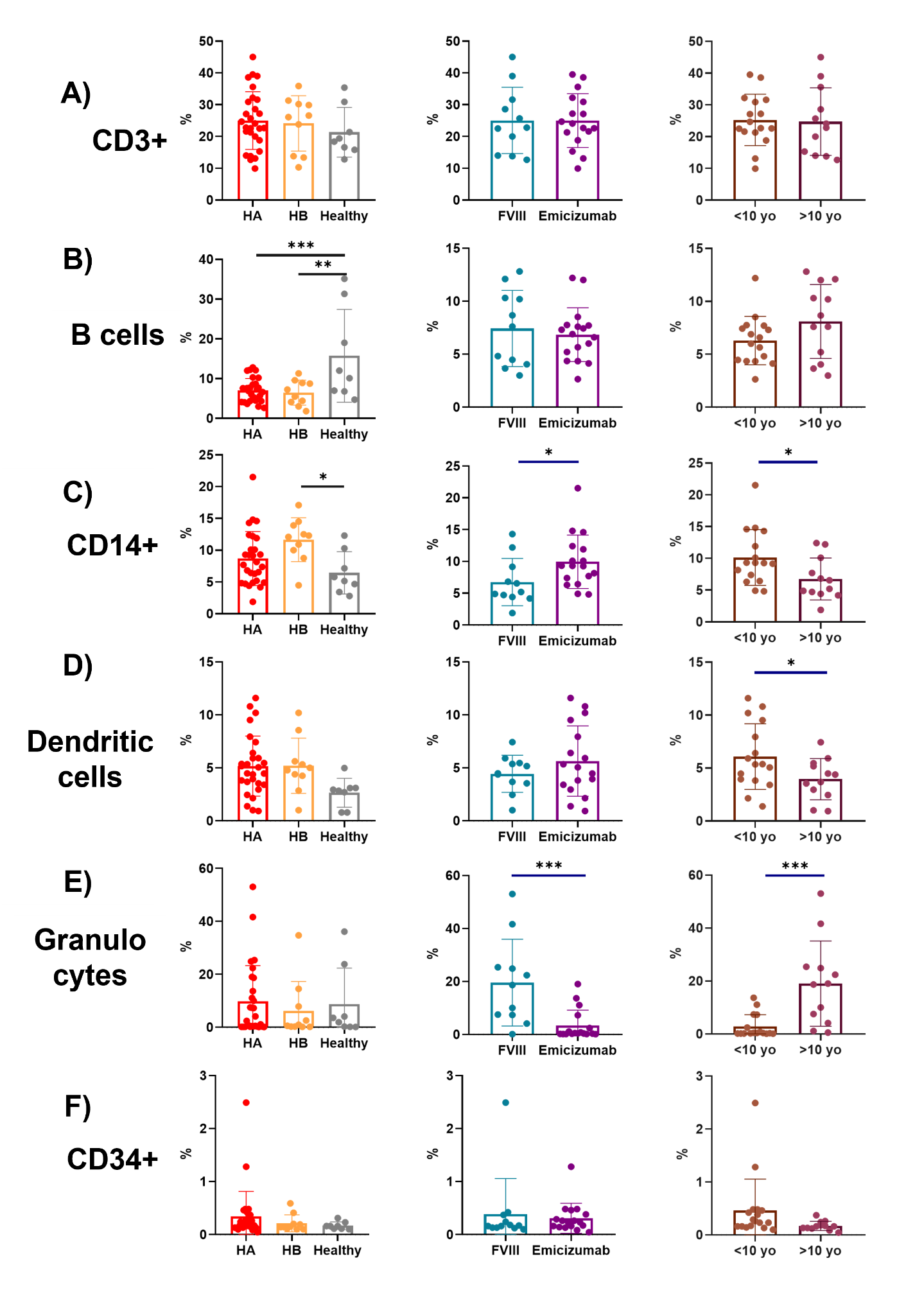


**Figure S2. Flow cytometry evaluation of all the blood immune populations analyzed expressed as % on live cells**. Comparison between HAvs HB patients, FVIII- vs Emicizumab-treated HA patients and <10 year- vs >10 year-old HA patients. A) Percentage of CD3+ cells. B) Percentage of B cells. C) Percentage of CD14+ cells. D) Percentage of dendritic cells. E) Percentage of granulocytes. F) Percentage of CD34+ cells. All data are represented as scatter plot with bar where the upper part of the bar is equal to the mean of the individual values belonging to the groups and the standard deviation (SD) is calculated. One-way ANOVA has been used for comparing the three groups, while unpaired t-test was used when two groups were compared. (p<0,05=*; p<0,01=**; p<0,001=***; p<0,0001=****)


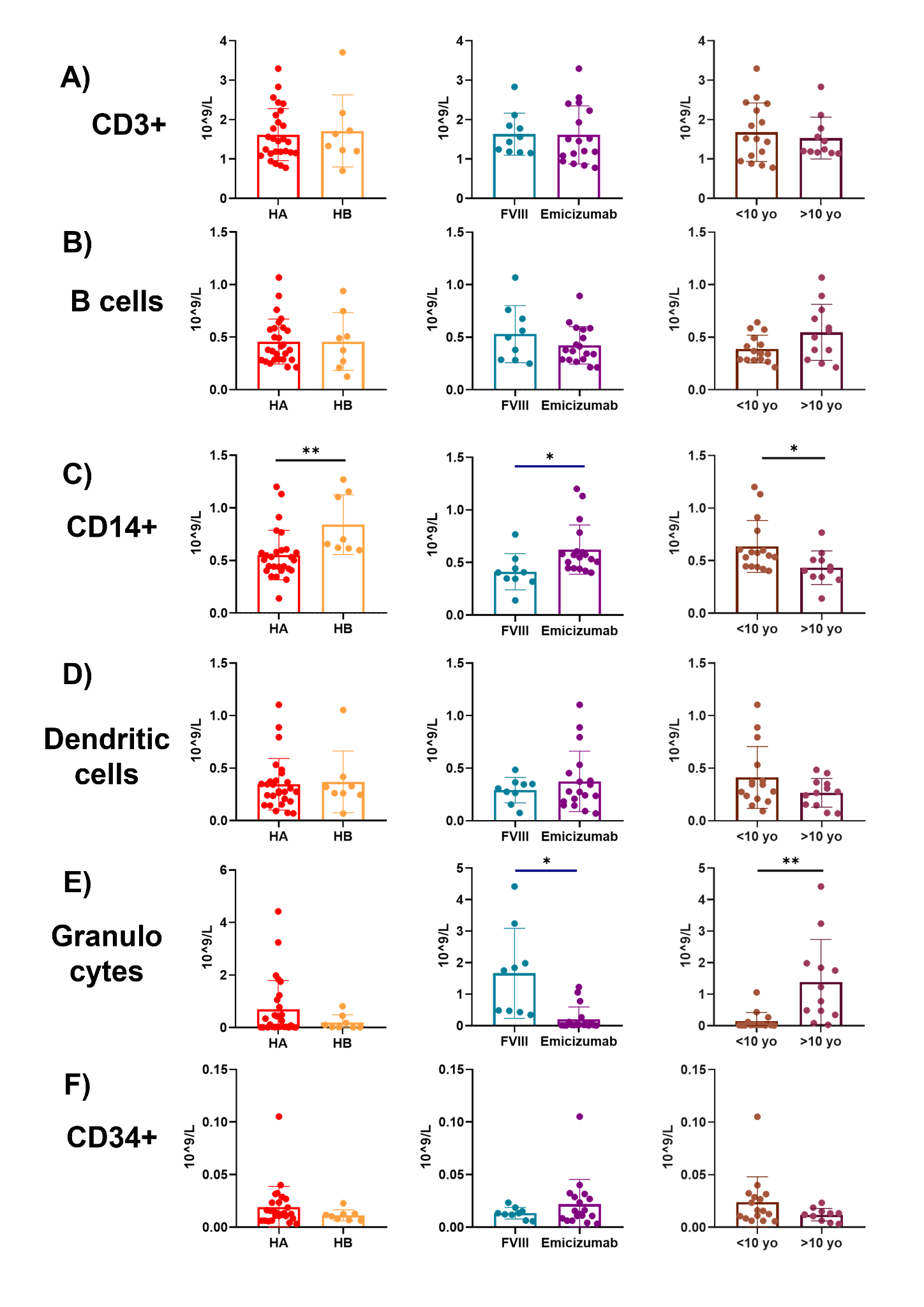


**Figure S3. Flow cytometry evaluation of all the blood immune populations analyzed expressed as number on live cells.** Comparison between HA vs HB patients, FVIII- vs Emicizumab-treated HA patients and <10 year- vs >10 year-old HA patients. The number/L of the analyzed cell populations were derived by multiplying the percentages obtained by flow cytometry with the corresponding white blood cell (WBC) counts, as determined from the total blood count performed on the day of sample collection for each patient. All data are represented as scatter plot with bar where the upper part of the bar is equal to the mean of the individual values belonging to the groups and the standard deviation (SD) is calculated. Unpaired t-test was used to compare the two groups. (p<0,05=*; p<0,01=**; p<0,001=***; p<0,0001=****)


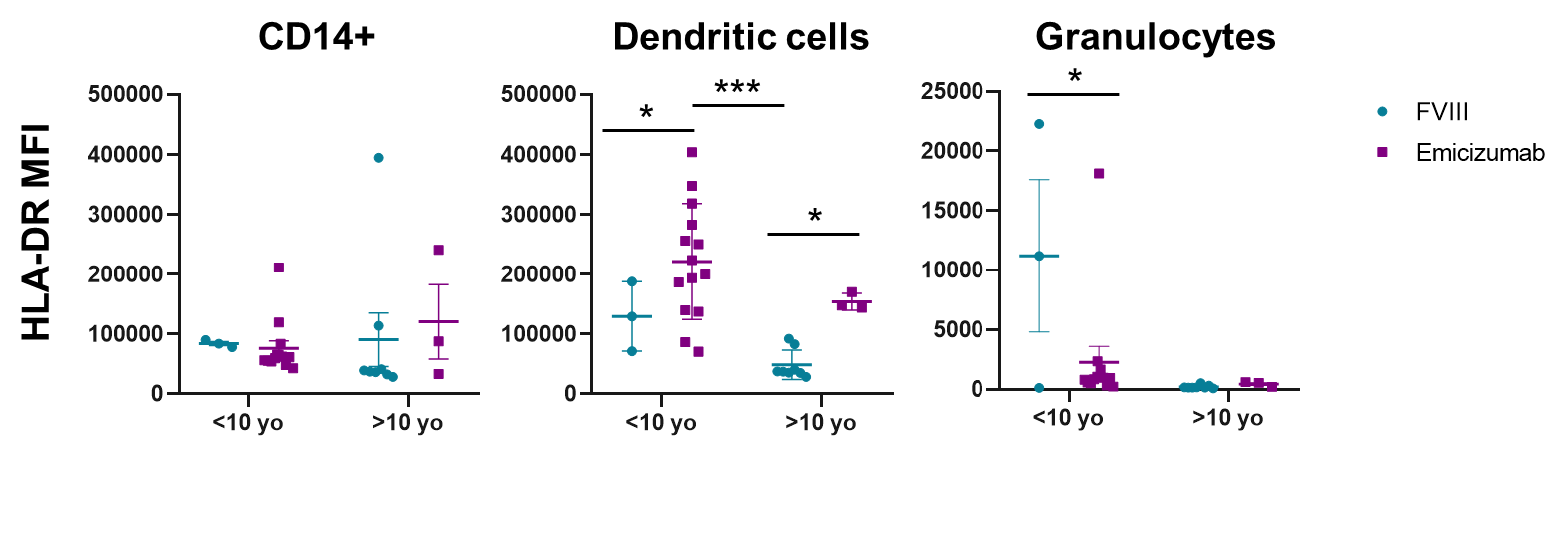


**Figure S4. Quantification of HLA-DR expression on blood immune cell surface through Median Fluorescent Intensity (MFI).** Stratification of HA patients according to their age (<10 years old and > 10 years old) and undergoing treatment, either rFVIII or Emicizumab. All data are represented as a scatter plot where the values for individual patients are represented by dots, while the middle line represents the means of the individual groups ± SD. Two-way ANOVA has been used for comparing the two groups. (p<0,05=*; p<0,01=**; p<0,005=***; p<0,0001=****)


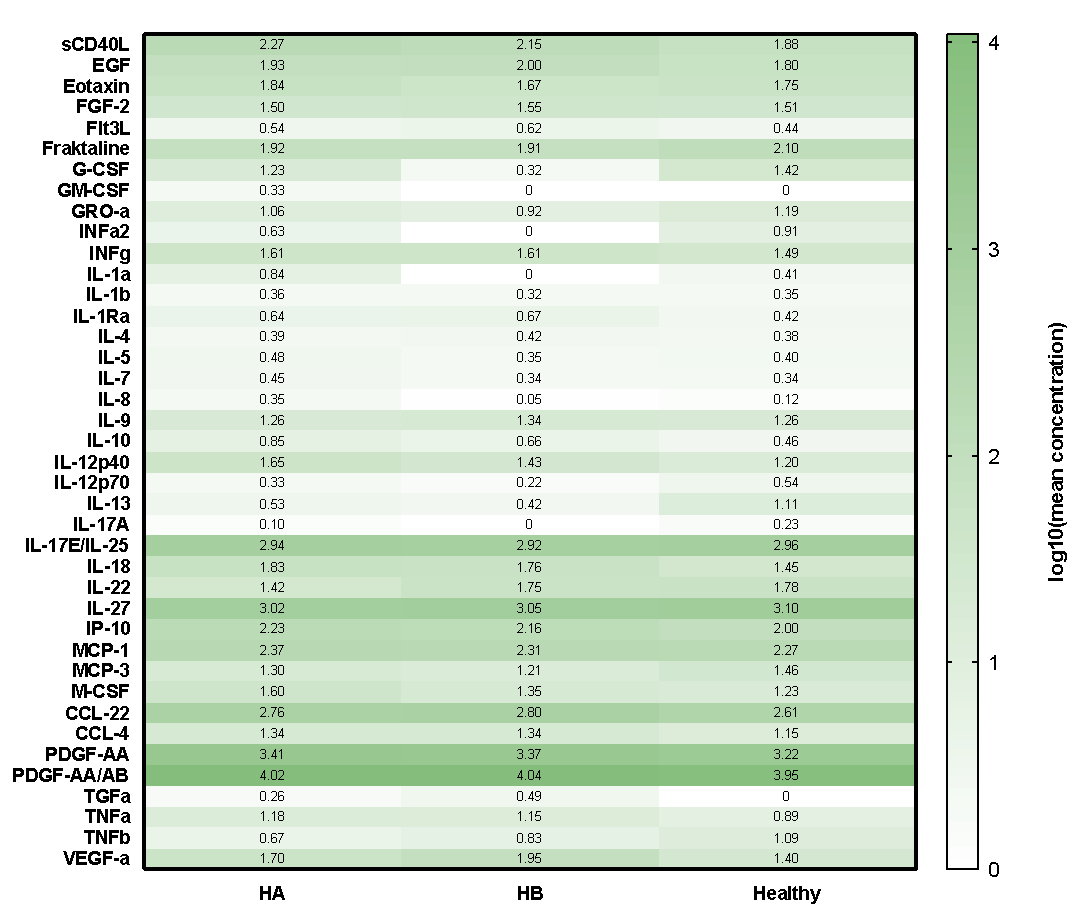


**Figure S5. Plasma cytokine comparison between HA (n=24), HB (n=8) and healthy (n=5) subjects.** Values are detected as concentration (pg/mL) for each sample and they are represented as a single-gradient heatmap as group’s log10 (mean value).


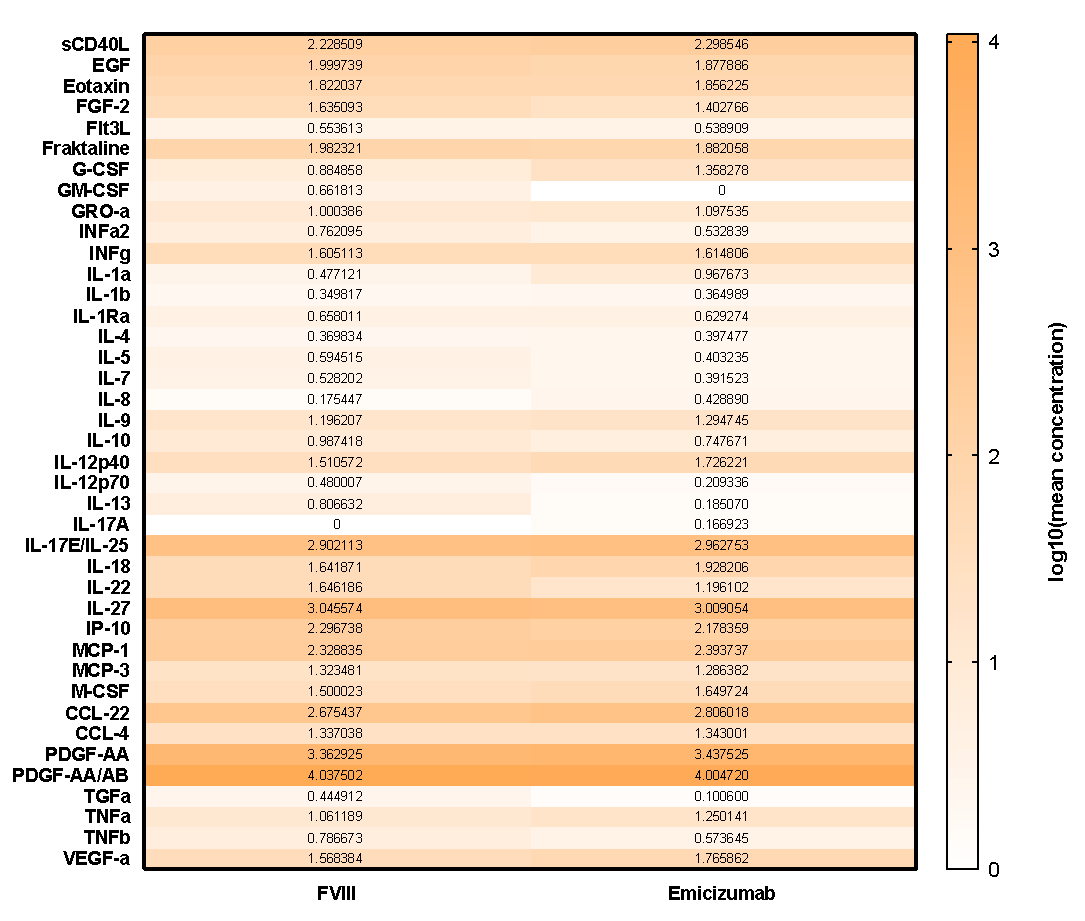


**Figure S6. Plasma cytokine comparison between HA patients in FVIII (n=8) or Emicizumab (n=15) prophylaxis**. Values are detected as concentration (pg/mL) for each sample and they are represented as a single-gradient heatmap as group’s log10 (mean value).


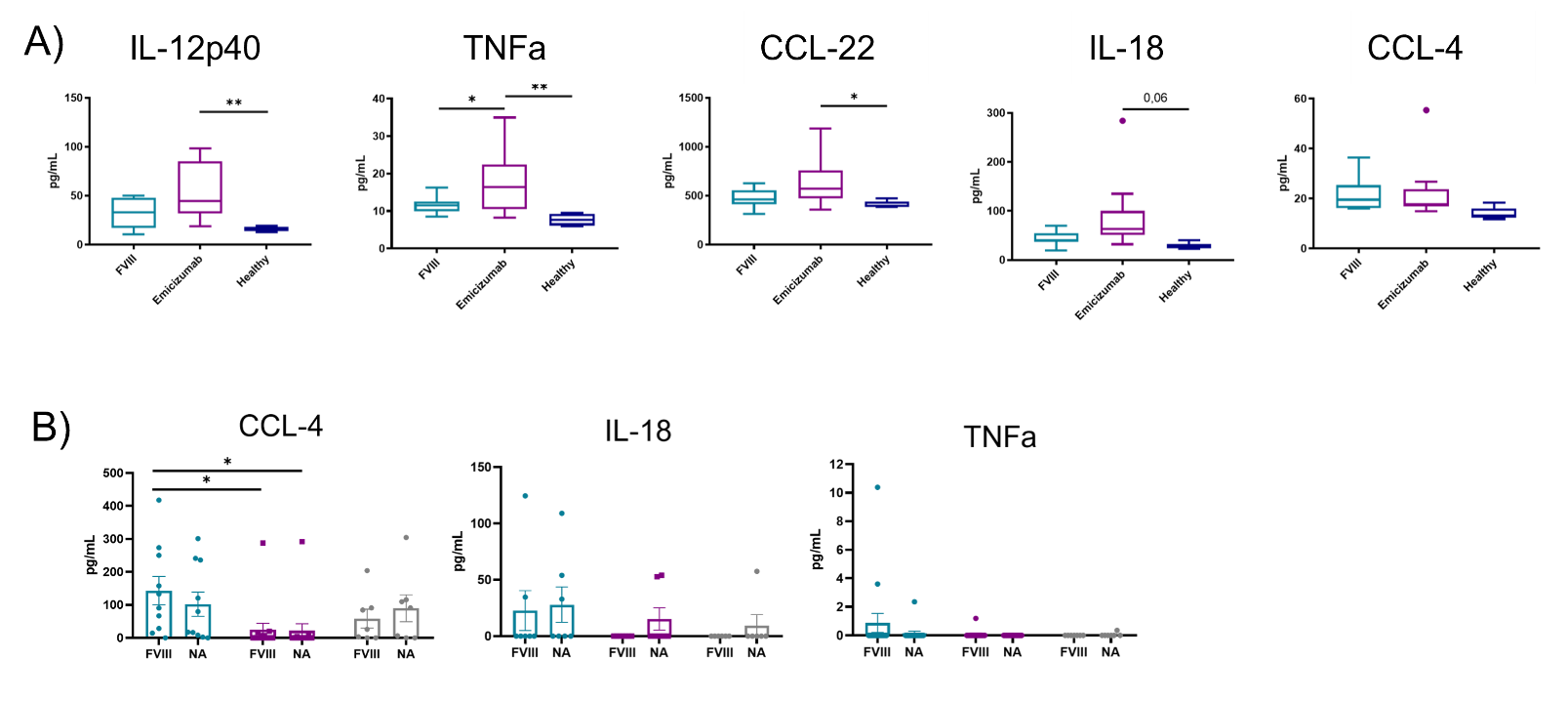


**Figure S7. Monocyte/macrophages-related cytokines**. A) Graphs showing comparison of IL12p40, TNF-a, CCL-22, IL-18 and CCL-4 quantification in plasma of HA patients stratified according to their undergoing treatment, either rFVIII or Emicizumab, and healthy donors. B) Graphs are showing CCL4, IL-18 and TNF-a expression by monocyte-derived macrophages from each group following FVIII stimulation (FVIII) compared to untreated (NA). Comparison between HA macrophages, stratified according to their undergoing treatment, either rFVIII or Emicizumab, and healthy macrophages. All data are represented as a scatter plot where the values ​​for individual patients are represented by dots, while the middle line represents the means of the individual groups ± SD. All undetected samples by the test were considered as 0 pg/mL. One-way ANOVA has been used for comparing the groups. (p<0,05=*; p<0,01=**; p<0,001=***; p<0,0001=****)

**Table S4. Group mean values of plasma cytokines in patients with HA, HB, and healthy controls.** The last column indicates statistical comparisons between groups, with symbols indicating significant differences at p<0.05: † = HA vs HB; ‡ = HA vs Healthy; § = HB vs Healthy; / = no difference.

| Cytokine | HA (n=24) pg/mL (± standard deviation) | HB (n=8) pg/mL (± standard deviation) | Healthy (n=5)  pg/mL (± standard deviation) | p-value (<0,05) |
| --- | --- | --- | --- | --- |
| sCD40L | 187,75 (223,50) | 142,77 (75,20) | 76,22 (53,10) | / |
| EGF | 84,66 (99,10) | 99,44 (57,23) | 63,64 (24,11) | / |
| Eotaxin | 69,78 (50,08) | 46,3 (13,07) | 56,21 (11,91) | / |
| FGF2 | 31,98 (19,72) | 35,24 (17,44) | 35,52 (9,94) | / |
| Flt3L | 3,50 (1,64) | 4,16 (2,14) | 2,79 (0,40) | / |
| Fraktalin | 83,64 (41,91) | 80,92 (20,14) | 125,42 (35,87) | ‡ |
| G-CSF | 17,14 (28,52) | 2,12 (4,63) | 26,5 (20,03) | / |
| GM-CSF | 2,16 (6,67) | 0 | 0,062 (0,14) | / |
| GRO-α | 11,58 (11,51) | 8,4 (5,24) | 15,38 (9,79) | / |
| INF-α2 | 4,3 (10,89) | 0 | 8,29 (8,53) | / |
| INF-γ | 40,85 (21,14) | 40,64 (13,91) | 30,84 (17,42) | / |
| IL-1α | 6,87 (25,72) | 0,07 (0,18) | 2,58 (2,86) | / |
| IL-1β | 2,29 (2,03) | 2,10 (1,26) | 2,25 (1,26) | / |
| IL-1Ra | 4,37 (2,36) | 4,64 (1,82) | 2,62 (0,92) | / |
| IL-2 | 0,18 (0,33) | 0,07 (0,12) | 0,36 (0,34) | / |
| IL-3 | 0,26 (0,42) | 0,13 (0,11) | 0,44 (0,25) | ‡ |
| IL-4 | 2,44 (1,81) | 2,62 (1,51) | 2,42 (1,32) | / |
| IL-5 | 3,06 (3,53) | 2,23 (1,44) | 2,51 (0,89) | / |
| IL-6 | 0,36 (0,58) | 0,68 (0,91) | 0,42 (0,31) | / |
| IL-7 | 2,81 (2,58) | 2,21 (0,88) | 2,19 (1,37) | / |
| IL-8 | 2,24 (3,79) | 1,13 (0,40) | 1,32 (0,48) | / |
| IL-9 | 18,21 (6,50) | 21,86 (10,80) | 18,43 (6,54) | / |
| IL-10 | 7,14 (10,04) | 4,55 (4,51) | 2,94 (1,91) | / |
| IL-12p40 | 44,56 (24,85) | 26,90 (13,98) | 16,02 (2,54) | ‡ |
| IL-12p70 | 2,14 (2,09) | 1,61 (2,08) | 3,46 (1,16) | ‡ |
| IL-13 | 3,36 (11,60) | 2,62 (7,41) | 12,97 (29,01) | / |
| IL-15 | 0,22 (0,41) | 0,06 (0,01) | 0,26 (0,21) | / |
| IL-17A | 1,25 (1,58) | 0,39 (0,52) | 1,69 (1,11) | / |
| IL-17E | 872,95 (523,40) | 828,25 (226,21) | 919,3 (679,32) | / |
| IL-17F | 1,51 (4,22) | 0 | 0 | / |
| IL-18 | 67,71 (53,31) | 57,59 (20,28) | 27,9 (6,88) | ‡ |
| IL-22 | 26,42 (32,70) | 55,88 (66,60) | 60,42 (113,20) | / |
| IL-27 | 1054,66 (352,10) | 1133,70 (271,28) | 1254,73 (699,96) | / |
| IP-10 | 168,50 (113,68) | 144,65 (82,98) | 101,18 (33,39) | / |
| MCP-1 | 234,70 (100,55) | 206,93 (119,45) | 188,77 (50,92) | / |
| MCP-3 | 19,98 (9,65) | 16,44 (4,61) | 28,91 (13,09) | § |
| M-CSF | 39,76 (45,02) | 22,40 (14,90) | 17,06 (7,60) | / |
| CCL-22 | 577,47 (194,14) | 637,13 (180,06) | 410 (37,84) | ‡ |
| CCL-4 | 21,91 (8,71) | 21,84 (7,76) | 14,00 (2,54) | ‡ |
| PDGF-AA | 2576,49 (1945,24) | 2323,72 (775,55) | 1674,56 (1023,03) | / |
| PDGF-AB/BB | 10419,40 (3561,29) | 10894,20 (1806,10) | 8926,52 (2467,38) | / |
| TGF-α | 1,83 (3,38) | 3,11 (4,71) | 0,29 (0,28) | / |
| TNF-α | 15,70 (6,76) | 14,06 (7,69) | 7,64 (1,57) | ‡ |
| TNF-β | 4,63 (3,25) | 6,83 (8,38) | 12,48 (16,29) | / |
| VEGF-α | 50,33 (32,54) | 88,71 (67,66) | 25,25 (11,46) | †,§ |
